# Neural Connectivity: How to Reinforce the Bidirectional Synapse Between Basic Neuroscience and Routine Neurosurgical Practice?

**DOI:** 10.3389/fneur.2021.705135

**Published:** 2021-07-01

**Authors:** Hugues Duffau

**Affiliations:** ^1^Department of Neurosurgery, Gui de Chauliac Hospital, Montpellier University Medical Center, Montpellier, France; ^2^Team “Plasticity of Central Nervous System, Stem Cells and Glial Tumors,” National Institute for Health and Medical Research (INSERM), U1191 Laboratory, Institute of Functional Genomics, University of Montpellier, Montpellier, France

**Keywords:** awake neurosurgery, brain mapping, neural connectivity, neuroscience, neuroplasticity

## Introduction

The quest for all neuroscientists is to better understand neural foundations underpinning human behavior. Neurosurgery offers a unique opportunity to be directly connected to the human connectome, and to provide further findings into brain processes—complementary to current investigations mainly relying on functional neuroimaging (FNI), comprising functional MRI, and diffusion tensor imaging (DTI) (1–4). Especially, awake patients with electrostimulation mapping (ESM) may benefit from an extensive neuropsychological assessment in real-time throughout surgery. This paradigm resulted in the description of the human homunculus by Penfield and Boldrey (5) almost one century ago and in the re-visitation of cortical organization of language by Ojemann (6). However, despite these pioneering works, the contribution of neurosurgery to fundamental neurosciences remains relatively modest, particularly in comparison with the numerous FNI reports (7, 8). Reversely, advances in brain connectomics, which led to new propose-built neurobiological models underlying neurocognition thanks to an improved knowledge of neural connectivity, have not (yet) extensively been incorporated in neurosurgical practice.

Here, the purpose is to consider solutions to reinforce the synapse, which should be bidirectional, between basic neuroscience and routine neurosurgery, in order to bring about synergies across research and clinical worlds based upon a dual vision of scientists and physicians.

## Contribution of Brain Surgical Mapping To Investigate Neural Connectivity

In the emerging field of connectomics, which aims at exploring neural connectivity, brain ESM during awake surgery provides direct insights into the function of both cortical structures and white matter tracts (WMT). Indeed, axonal ESM of the subcortical fibers may elicit a transient disruption of the functional network sub-served by the bundle stimulated, and consequently, may generate a specific behavioral deficit which immediately resolves when ESM stops (9). Beyond a transitory dys-synchronization within a discrete critical circuit, ESM can also disrupt between-network inter-communication, resulting in multi-tasking disorders, e.g., the patient is still able to move or to speak separately, but cannot do both simultaneously (10). Such original anatomo-functional correlations gained from on-line intraoperative cortico-subcortical ESM led researchers to re-visit the functional connectivity mediating neural systems, such as movement execution and control (11), oral and written language (12, 13), semantics (14), executive control (15), self-evaluation (16), or theory of mind (17, 18). Interestingly, this ESM permitted a reappraisal of the model of the human connectome (19), and proposed a new theory relying on a meta-network (network of networks) organization of the brain, i.e., with perpetual changes of intra- and across circuit interactions allowing adapted behavior (20).

Moreover, longitudinal ESM explorations, particularly based upon serial mapping in patients who underwent several awake surgeries because of tumor relapse (21), enabled researchers to better understand mechanisms sustaining neuroplasticity (22). Remarkably, optimal recovery following massive brain resections in tumor patients, with normal scores on objective neuropsychological assessments of conation, cognition, and emotion despite surgery within structures classically deemed “eloquent” according to localizationist dogma, evidenced a considerable potential of neural configuration, higher than previously thought (23, 24). Nonetheless, the limitations of this plastic potential have also been demonstrated, by emphasizing the critical role of the subcortical connectivity (25). Thus, a “minimal common brain” has been suggested, with a low level of inter-individual variability and a low power of functional compensation after cerebral injury (26): such a “cerebral skeleton” is mainly constituted by WMT (27).

Neurosurgery represents a gold mine to develop innovative models in cognitive neurosciences, thanks to the direct information about neural connectivity collected into the operating room (OR) by means of ESM (19). It is regrettable that these original data which challenged the obsolete model of localizationism, e.g., by evidencing that Broca's area was non-critical for speech, were neglected for a long time by neurologists and neuroscientists (28). A solution to reunify learned societies can be to combine findings provided by ESM with those gained from FNI in healthy volunteers as well as before and after surgery in brain-damaged patients (22). Based upon different backgrounds and complementary areas of expertise (neuroanatomy, neuroimaging, awake brain mapping, cognitive neuroscience, neurophysiology, neurocomputational modeling, etc.), speaking a universal language may allow researchers to understand more rapidly and accurately neurobiology-sustaining complex human behavior. Reversely, an improved knowledge of brain circuitry could be helpful in neurosurgical practice, to optimize postoperative outcomes.

## How To Integrate A Better Understanding of The Cerebral Connectome into Neurosurgical Practice?

For many decades, neurosurgeons mainly paid attention to the cortex, with few considerations regarding subcortical connectivity. Beyond research purposes, recent advances in DTI resulted in an improved investigation of WMT for surgical planning (29, 30). Moreover, preoperative tractography data were incorporated into neuronavigational systems to better define surgical approach and limits of resection into the OR (31, 32). However, even though these technological developments played a major role in basic research to explore the connectome (3, 8), and started to bring the gap between neuroscience and clinical applications, the link across both worlds is still weak and superficial. Indeed, the majority of neurosurgeons has no background in FNI methodology and has the wrong belief that DTI is a reliable insight into WMT function. Yet, despite a growing excitement in imaging-guided neurosurgery, these techniques intrinsically suffer from major limitations (from the data acquisition to the statistical models used) (33, 34), the main one being that tractography does not provide any information about the function of subcortical fibers—but is only an indirect reflection of their structures (35). Therefore, an abusive surgical use of these methods whose pitfalls are poorly controlled, even if initially designed to help neurosurgeons, may paradoxically become dangerous. For example, FNI may result in a non-selection for surgery while the tumor could have been removed (with a loss of chance from an oncological perspective), or conversely, may lead to the resection being pushed too far by cutting critical pathways not identified as essential by DTI (with some loss likely from a functional perspective) (36). Furthermore, although FNI is a fantastic didactic tool to help junior neurosurgeons to build an accurate 3D representation of structural and functional connectivity in their own mental imagery, especially when combined with dissection in specimens (37, 38), the overuse of this technology for brain surgery may lead to the opposite effect, i.e., to an addiction to FNI, preventing an optimal surgical act if this tool was unavailable (36).

To reinforce the synapse with fundamental research, neurosurgeons should be neuroscientists, since he/she also has the responsibility to explore the connectome by him/herself, with the ultimate goal of improving postoperative outcomes. Thus, besides a better comprehension of the potentials and drawbacks of FNI for its more appropriate utilization, intraoperative ESM should be used more systematically (39). In addition to cortical mapping, axonal ESM is the sole methodology enabling a direct study of WMT function, concerning one specific bundle and interplay between neural circuits (10). Awake mapping led to a paradigmatic shift from image-guided resection to functional-guided resection, especially in neuro-oncology (40). Remarkably, awake ESM guiding the resection until the individual eloquent cortico-subcortical networks have been encountered resulted in an improvement of both neurological and oncological outcomes (41). Functionally speaking, a connectome-based resection allowed for a significant decrease of neurological morbidity (42–44) with preservation of conation, language, and higher-cognitive functions (e.g., complex movement control, verbal and non-verbal semantics, executive functions, mentalizing or metacognition) (45–48) making it possible to resume a normal life, including return to work in 97% of patients (49)—even for tumors involving areas presumed to be “eloquent” in a rigid localizationist framework (23, 24). Oncologically speaking, functional mapping-guided surgery has enabled a significant increase of extent of resection and overall survival, both in low-grade and high-grade gliomas (50–53). Yet, although ESM is now the gold standard for glioma surgery (39), it is still underused in neurosurgery in general.

This concept of connectome-based surgery represents an actual solution to introduce more robust neuroscience into the OR. Firstly, beyond its participation in the development of original models of neurocognition (12, 13, 19, 20), awake ESM also enabled the elaboration of a human atlas of neuroplasticity (25) and an atlas of critical cortico-subcortical networks (54–56). This can be helpful for neurosurgeons to predict whether the patient will recover or not according to the extent of resection, especially by highlighting the structures with a low potential of reorganization, such as the subcortical connectivity—the so-called “minimal common brain” mentioned above (27). To facilitate presurgical planning by predicting the distribution of essential neural connections to be preserved, a tool has recently been proposed for a practical use: it allows automated alignment of the cortico-subcortical maps of this probabilistic atlas with T1-weighted MRI of a given patient (57). These data can also be used to estimate before surgery the extent of resection thanks to a probabilistic map of tumor resectability, computed on the basis of postoperative residual glioma voluntarily left because of invading critical structures identified by intraoperative ESM (58).

Secondly, these new cognitive models of meta-networking organization of brain functions (12, 13, 19, 20) and the new atlases built based upon ESM (25, 27, 54–57) represent unmatched educational tools for neurosurgeons to learn 3D neural connectivity. Indeed, they provide real structural-functional information collected intraoperatively in patients who underwent awake surgery, and not virtual data as given by FNI, with different reconstructions according to the biostatistical modeling employed.

Thirdly, besides neuro-oncology, application of the concept of connectome-based resection relying on a better understanding of dynamic interplay within and across neural networks, may be considered in other fields of brain surgery. Surprisingly, although awake ESM was initially developed in epilepsy surgery (5, 6, 59), most of the series dedicated to non-tumoral epilepsy did not use intraoperative mapping in the modern literature. Yet, recent publications supported again the positive role of awake resection for epilepsy, notably with mapping of the subcortical pathways, (such as optic tracts to avoid visual field deficits), in a connectome paradigm of brain processing (60, 61)—knowing that the mechanisms of neuroplasticity are not similar in lesional vs. non-lesional epilepsy (62). An improved exploration of neural connectivity is also valuable for surgical approaches to deep lesions located within hard-to-reach areas. Typically, a trans-cortical approach to have access to the insula should take into account the sub-opercular connectivity, particularly the frontal part of the inferior fronto-occipital fasciculus (IFOF) and of the superior longitudinal fasciculus/arcuate fasciculus (AF) complex (63). Similarly, a transcortico-subcortical approach to the left posterior Medio basal region necessitates the knowledge, detection, and preservation of relevant WMT including the optic pathways, AF, IFOF, and inferior longitudinal fasciculus (64). Also, for deep cavernomas, the best surgical corridor through the subcortical fibers should be defined by validating in real-time that the neural connectivity crossed to reach the lesion was not crucial for brain functions (65). This is especially valuable for lesions involving a neural crossroad, e.g., the ventral precentral fiber intersection area (66) or the temporoparietal fiber intersection area traversed by seven WMT (67). Finally, such a 3D mental representation in the brain's neurosurgeon is also useful for emergent surgery, such as the evacuation of a left deep temporo-insular hematoma under general anesthesia, in an aphasic patient with mass effect. If the patient remained aphasic after surgery, it could be thought that this was related to an irreversible damage generated by the hematoma, whereas in some cases, it might be due to a traumatic surgical corridor through the critical fibers, such as the IFOF—meaning that the patient could have recovered if the approach would have been modified thanks to better knowledge of the connectome, even in asleep patients. To this end, fiber dissection in cadavers to accelerate the learning curve is of utmost importance.

## Perspectives

It is time to overcome the divide between fundamental research in neurosciences, increasing reliance on FNI and neurocomputational modeling which usually do not take into account the structural constraints, and the neurosurgical routine which should preserve the neural connectivity under penalty of no postoperative recovery, but which can also propose new cognitive models based upon direct observation of the connectome into the OR. Dual training for juniors, at the start of their courses, would enhance their chance to create reciprocal connections across basic and clinical neuroscience, and to develop more translational research in their daily practice dedicated to brain understanding and restoration.

## Author Contributions

The author confirms being the sole contributor of this work and has approved it for publication.

## Conflict of Interest

The author declares that the research was conducted in the absence of any commercial or financial relationships that could be construed as a potential conflict of interest.
